# Using Dispensing Data to Evaluate Adherence Implementation Rates in Community Pharmacy

**DOI:** 10.3389/fphar.2019.00130

**Published:** 2019-02-26

**Authors:** Andrea Torres-Robles, Elyssa Wiecek, Rachelle Cutler, Barry Drake, Shalom I. Benrimoj, Fernando Fernandez-Llimos, Victoria Garcia-Cardenas

**Affiliations:** ^1^Graduate School of Health, University of Technology Sydney, Sydney, NSW, Australia; ^2^Faculty of Engineering and Information Technology, University of Technology Sydney, Sydney, NSW, Australia; ^3^Department of Social Pharmacy, Faculty of Pharmacy, University of Lisbon, Lisbon, Portugal

**Keywords:** big database, dispensing records, medication adherence, community pharmacy, adherence implementation

## Abstract

**Background:** Medication non-adherence remains a significant problem for the health care system with clinical, humanistic and economic impact. Dispensing data is a valuable and commonly utilized measure due accessibility in electronic health data. The purpose of this study was to analyze the changes on adherence implementation rates before and after a community pharmacist intervention integrated in usual real life practice, incorporating big data analysis techniques to evaluate Proportion of Days Covered (PDC) from pharmacy dispensing data.

**Methods:** Retrospective observational study. A de-identified database of dispensing data from 20,335 patients (*n* = 11,257 on rosuvastatin, *n* = 6,797 on irbesartan, and *n* = 2,281 on desvenlafaxine) was analyzed. Included patients received a pharmacist-led medication adherence intervention and had dispensing records before and after the intervention. As a measure of adherence implementation, PDC was utilized. Analysis of the database was performed using SQL and Python.

**Results:** Three months after the pharmacist intervention there was an increase on average PDC from 50.2% (SD: 30.1) to 66.9% (SD: 29.9) for rosuvastatin, from 50.8% (SD: 30.3) to 68% (SD: 29.3) for irbesartan and from 47.3% (SD: 28.4) to 66.3% (SD: 27.3) for desvenlafaxine. These rates declined over 12 months to 62.1% (SD: 32.0) for rosuvastatin, to 62.4% (SD: 32.5) for irbesartan and to 58.1% (SD: 31.1) for desvenlafaxine. In terms of the proportion of adherent patients (PDC >= 80.0%) the trend was similar, increasing after the pharmacist intervention from overall 17.4 to 41.2% and decreasing after one year of analysis to 35.3%.

**Conclusion:** Big database analysis techniques provided results on adherence implementation over 2 years of analysis. An increase in adherence rates was observed after the pharmacist intervention, followed by a gradual decrease over time. Enhancing the current intervention using an evidence-based approach and integrating big database analysis techniques to a real-time measurement of adherence could help community pharmacies improve and sustain medication adherence.

## Introduction

Medication non-adherence remains a major burden on the health care system. Estimated annual costs of non-adherence range between $949 and $44,190 per patient (Cutler et al., 2018), up to $300 billion in the United States in avoidable funds (Institute, 2009) and €125 billion annually to the European Union (Pharmaceutical Group of the European Union, 2018). As a result, various interventions in diverse settings have shown marginal improvements in medication adherence (Nieuwlaat et al., 2014; Conn and Ruppar, 2017). However, in order to further progress the enhancement of non-adherence, we must fully understand and correctly utilize measures of adherence depending on the purpose or design of the study (Lehmann et al., 2014). The accurate and timely measurement of medication adherence is not only crucial to provide better evidence but creates problematic and expensive consequences if performed incorrectly (Lam and Fresco, 2015).

Multiple methods and tools are available for measuring adherence but guidance for the most suitable measure for healthcare professionals and researchers is still lacking (Whalley Buono et al., 2017). Moreover, measures of adherence must also take into consideration the different components of the medication taking process as recently defined by the ascertaining barriers to compliance (ABC) taxonomy. The medication taking process begins at initiation of treatment, continues at implementation or the extent to which a patient’s actual dosing corresponds to the prescribed dosing regimen, and persistence or the time from initiation to discontinuation. These components all individually carry significant insight into patient medication-use behavior (Vrijens et al., 2012).

An increase in the accessibility of health system data and advancements in electronic information of medication use has permitted new insight into patients’ medication behavior (Whalley Buono et al., 2017). The increased availability of big data in health has enabled the utilization of quality performance measurement across various aspects. Specifically in pharmacy, large data sets of prescription dispensing information, also known as pharmacy claims or prescription refill data, have become more readily available from the ease of electronic information, making it useful for analyzing medication adherence (Raebel et al., 2013) and providing a viable and economical approach for its estimation in real time (Vik et al., 2004). Even in the absence of a gold standard, the use of dispensing data has been a staple in adherence measurements due to their validity, relative accessibility and inexpensiveness (Simons et al., 2008; Martin et al., 2009; Greevy et al., 2011; Arnet et al., 2014; Holdford and Saxena, 2015), creating valuable data sets (Blaschke et al., 2012; Ma et al., 2015). Validated and endorsed by the Pharmacy Quality Alliance and having been used for over two decades, examples of dispensing data’s use in the literature are abundant and increasingly frequent (Martin et al., 2009; Pillittere-Dugan et al., 2009). This allows the calculation of measures of adherence such as Medication Possession Ratio (MPR) and Proportion of Days Covered (PDC), two validated measures of adherence based on the percentage of days the patient has medication available. While difficult to measure in previous traditional methods, dispensing data creates an easier system in which to evaluate and monitor all stages of the medication-use process (Blaschke et al., 2012). From this, long-term patterns can be identified and evaluated which before were often not feasible in randomized controlled trials investigating adherence due to short durations. This might also be essential in order to monitor the long-term effectiveness of medications during their post-authorization phase.

Frequently revealed in long-term monitoring are declining trends in adherence, indicating the issue of maintaining adherence over time as crucial as improving adherence at a cross-sectional time point (Cooper et al., 2011; Blaschke et al., 2012; Demonceau et al., 2013). Instant feedback during the dispensing process can allow the monitoring of patient adherence in real-time, especially by community pharmacists, and therefore, trigger adherence interventions when suboptimal adherence levels are identified (Sodihardjo-Yuen et al., 2017). Interventions to improve medication adherence in research projects delivered by community pharmacists have been shown to be effective (Nieuwlaat et al., 2014; Milosavljevic et al., 2018). This evidence has usually been generated through clinical trials, conducted in well-defined and controlled environments. However, whether these trials produce results that are applicable to everyday practice and whether the effects are maintained in real-life settings usually remains unknown. In real-life practice, patients are often exposed to community pharmacist interventions during the dispensing of medicines but no analysis of the impact of the intervention on improving adherence long-term is usually conducted. Retrospective observational designs and pragmatic trials can include measures of adherence from dispensing data that allow evaluation of the effectiveness of these interventions in real life environments (Whalley Buono et al., 2017). The objective of this study was to use big data techniques on pharmacy dispensing data to analyze the effectiveness of a community pharmacist-led intervention on medication adherence implementation in patients using rosuvastatin, irbesartan and/or desvenlafaxine in Australia. With this study, we were able to both evaluate an intervention’s long-term effect on improving adherence in addition to evaluating a big data approach and methodology to analyzing adherence implementation rates.

## Materials and Methods

Retrospective observational study of dispensing records of patients receiving a real-life educational-based intervention to enhance medication adherence from community pharmacists across Australia.

### Pharmacist Intervention

GuildLink Pty Ltd is part of a group of companies which is wholly owned by the Pharmacy Guild of Australia and provides software solutions to community pharmacies in Australia for documenting the provision of diverse pharmacy services. Their MedScreen Compliance Program targets non-adherent patients when a calculated MPR is below 70%, alerting the dispensing pharmacist to offer an educational-based intervention aiming at improving medication adherence. A guided interaction between the pharmacist and patient is then offered which encompasses the following steps: (1) exploration and identification of real or perceived barriers to medication adherence, (2) provision of patient education on proper medication use in an oral or written (patient handouts about medicines information) manner and the importance of adherence, (3) goal-setting for their treatment targets, and (4) recording of the interaction. Patients could receive one or multiple interventions across multiple time periods depending on the calculated MPR, alerting the pharmacist to invite the patient to the intervention if they remain below the 70% threshold.

### Data Source and Patients

GuildLink Pty Ltd GuildCare Software Databases were used for this study to assess dispensing data from Australian pharmacies that participate in the MedScreen Compliance programs. These databases contained de-identified primary care prescription data, dispensing data, and pharmacist intervention data from the affiliated pharmacies. Dispensing data (1 year before and after the first pharmacist’s intervention) for patients taking desvenlafaxine, irbesartan and/or rosuvastatin who had received the intervention previously described, was analyzed. No process indicators to validate the fidelity of the intervention were available as it was a real-life intervention. In order to calculate adherence implementation rates from dispensing data, two main exclusion criteria applied. The database did not record days’ supply for each individual dispense. Due to this, we assumed a once daily prescribed dose and therefore excluded patients with a prescribed quantity of less than 28 or more than 30 doses per dispense. In addition, more than two dispensing fills were needed to accurately calculate an adherence rate. This excluded patients with less than two dispensing dates before and after the intervention.

### Outcome: Adherence Implementation

Adherence implementation rates were calculated using the PDC. This indicator accounts for overlapping days’ supplied to allow a more conservative estimate of adherence and has been previously validated (Pillittere-Dugan et al., 2009). We selected it over MPR due to MPR’s overestimating effects when analyzing multiple medications and overlapping days.

### Data Analysis

The data was analyzed by integrating SQL (Microsoft SQL Server Management Studio Version 14.0.17213.0), Python (Version 2.7.14) and PyCharm (Version 2017.3.4, Community Edition) language programs to organize and retrieve the results.

In order to organize the final data table with the required components to be analyzed, some validations were performed. A unique Australian identifier code (PBSCode) linked to each script was used to infer missing quantity prescribed data per patient and organize the scripts corresponding to each drug. This code is always associated to a drug and a quantity to be prescribed, making it feasible to be used to infer missing quantities.

Analysis was conducted per trimesters, 1 year before and 1 year after the first pharmacist intervention, calculating the average PDC (%) and standard deviation (SD) for all the patients in each period of time using descriptive statistics. An additional sub-analysis regarding the number of adherent patients was performed. Cut-off for optimal adherence was defined as PDC equal or higher than 80% as this has been found to be reasonable for predictable hospitalizations in chronic diseases (Karve et al., 2009).

A sensitivity analysis was performed on patients who claimed the initial dispensing and their corresponding repeats (number of times an original prescription can be claimed in a pharmacy in Australia) to observe if there was a difference on the trend compared to the general analysis regardless of the repeat dispensing sequence.

### Ethics Statement

University of Technology Sydney Human Research Ethics Committee (HREC) approved this study (approval number ETH18-2312). The study was classified as having Nil/Negligible Risk. No personal or confidential data was included in the database. Therefore no informed consent was required.

## Results

### Study Sample

The database contained de-identified data of 2,530,562 patients from 3,318 community pharmacies across different states in Australia from 2014 to 2017. A total of 1,805 pharmacies across seven states in Australia and 20,335 patients (*n* = 11,257 using rosuvastatin, *n* = 6,797 on irbesartan, and *n* = 2,281 on desvenlafaxine) met the inclusion criteria and were included in the analysis. The average number of patients per pharmacy was 8.59 (SD: 5.14).

The distribution of patients according to gender was 56% female and 44% male of patients taking rosuvastatin, 61% female and 39% male on irbesartan and 70% female and 30% male on desvenlafaxine. Average age was 65 (SD: 11.76) in patients using rosuvastatin, 67 (SD: 12.42) in irbesartan and 50 (SD: 15.70) for desvenlafaxine.

### Implementation Adherence – PDC

**FIGURE 1:**
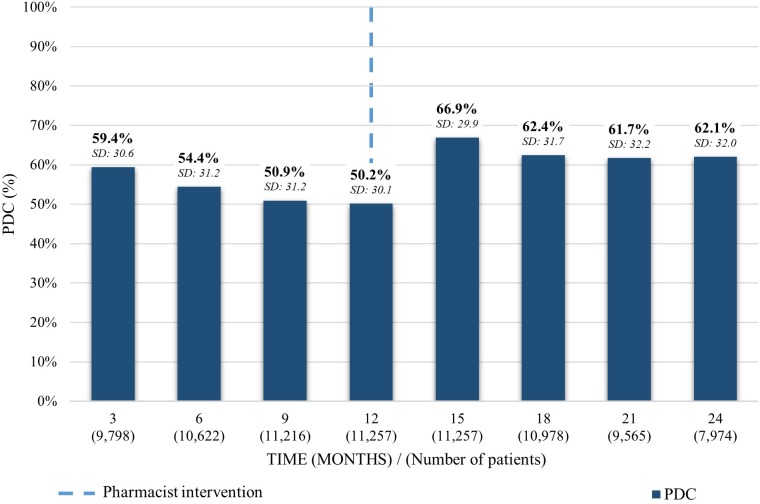
Average PDC before and after the pharmacist intervention for patients taking rosuvastatin. Analysis performed in each trimester 12 months before and 12 months after the first intervention. Average PDC and Standard Deviation (SD) reported.

The average PDC of patients taking rosuvastatin 12 months previous to the pharmacist intervention was 59.4% (SD: 30.6) decreasing on 9.2% to 50.2% (SD: 30.1) in the last trimester before the intervention. An increase of 16.7% was observed in the 3 months following the pharmacist intervention, reaching a 66.9% (SD: 29.9) average PDC, dropping to 62.1% (SD: 32.0) during the 12 months after the intervention (Figure 1).

**FIGURE 2:**
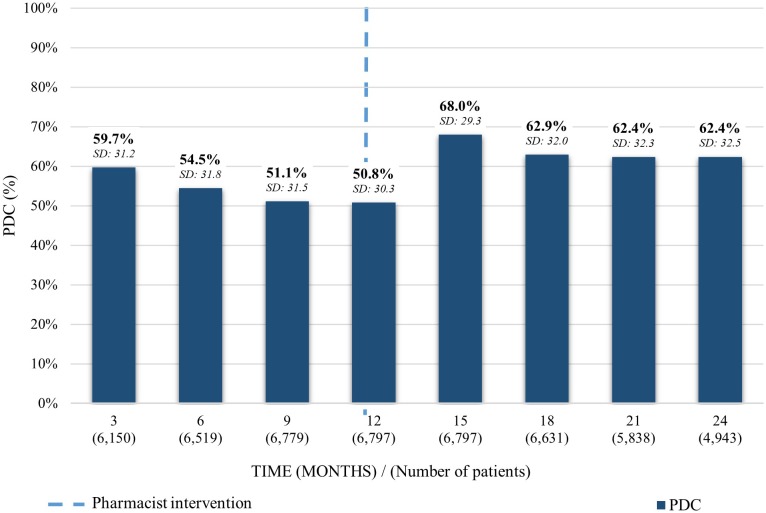
Average PDC before and after the pharmacist intervention for patients taking irbesartan. Analysis performed in each trimester 12 months before and 12 months after the first intervention. Average PDC and Standard Deviation (SD) reported.

For patients taking irbesartan, a gradual decrease of the average PDC was depicted over a 1-year period from 59.7% (SD: 31.2) to 50.8% (SD: 30.3). 3 months after the pharmacist intervention it increased 17.2% to an average PDC of 68.0% (SD: 29.3). Finally, it decreased 4.8% 12 months after the intervention to 62.4% (SD: 32.5) (Figure 2).

**FIGURE 3:**
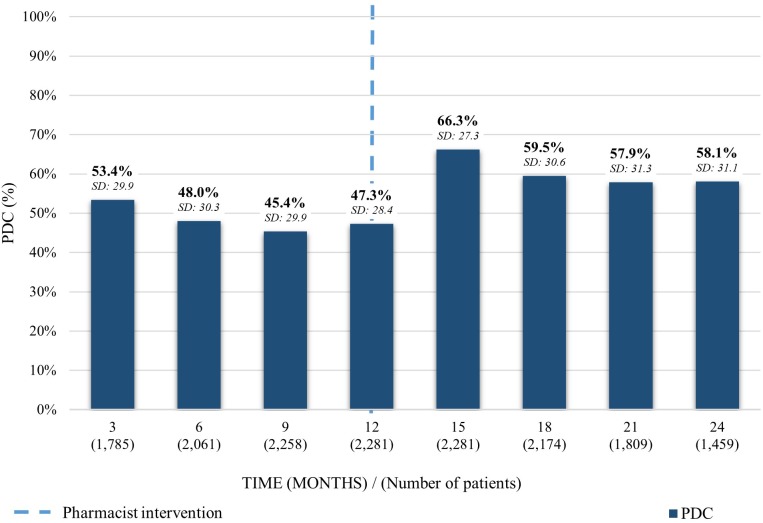
Average PDC before and after the pharmacist intervention for patients taking desvenlafaxine. Analysis performed in each trimester 12 months before and 12 months after the first intervention. Average PDC and Standard Deviation (SD) reported.

As for the average PDC on patients taking desvenlafaxine, a similar trend to the previous medications was observed. The PDC average declined on the first 12 months previous to the pharmacist intervention from 53.4% (SD: 29.9) to 47.3% (SD: 28.4). After the intervention, it increased 19% to 66.3% (SD: 27.3) and decreased 8.2% in the following 12 months to a PDC of 58.1% (SD: 31.1) (Figure 3).

Sensitivity analysis performed on patients claiming all dispensings in the affiliated pharmacies resulted in a similar trend with the PDC average increasing 15.7% from 59.0% (SD: 27.3) to 74.7% (SD: 27.7) after the pharmacist intervention and declining over the following 12 months on 9.6% to 65.1% (SD: 24.6).

### Sub-Analysis – Proportion of Adherent Patients

**FIGURE 4:**
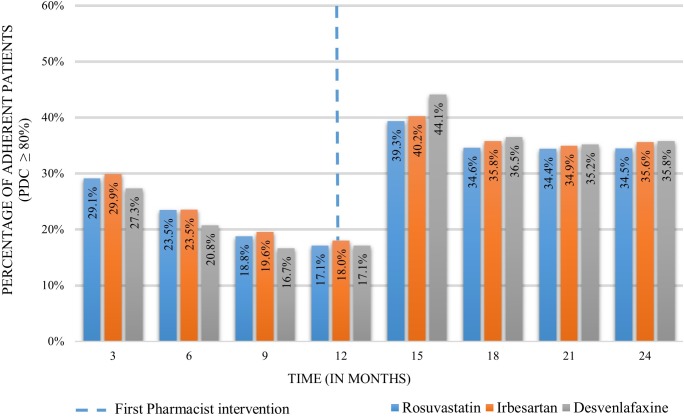
Proportion of adherent patients 12 months before and 12 months after the pharmacist intervention.

The proportion of adherent patients 12 months before performing the intervention was 29.1% (*n* = 2,851 patients), 29.9% (*n* = 1,838) and 27.3% (*n* = 488) for rosuvastatin, irbesartan and desvenlafaxine, respectively. These percentages decreased along the first year of analysis before the intervention to 17.1% (*n* = 1,927), 18.0% (*n* = 1,223), and 17.1% (*n* = 391) before providing the intervention. An increase was observed 3 months after the first intervention with a proportion of 39.3% (*n* = 4,428), 40.2% (*n* = 2,734), and 44.1% (*n* = 1,006). Twelve months after the intervention, the proportion of adherent patients diminished to 34.5% (*n* = 2,750) for rosuvastatin, 35.6% (*n* = 1,761) for irbesartan and 35.8% (*n* = 522) for desvenlafaxine (Figure 4).

## Discussion

Big database analysis techniques were integrated to analyze the dispensing data of 20,335 patients across community pharmacies in Australia receiving an educational-based adherence intervention prompted by the dispensing software when an MPR below 70% was identified. Data was analyzed from 1805 different community pharmacies, which represents 31.9% of all community pharmacies across Australia (The Pharmacy Guild of Australia, 2018). Records of 12 months before and 12 months after a pharmacist intervention were included, allowing the use of “real-world” data to estimate medication implementation adherence for three drugs (rosuvastatin, irbesartan and desvenlafaxine) over time.

Trends observed before and after the intervention in each of the drugs showed: (1) a gradual decrease in average PDC rates during a 1 year pre-intervention, (2) an increase after the pharmacist’s intervention was delivered, followed by (3) a subsequent decrease over time. This is consistent with previous evidence, which highlights the dynamic nature of medication adherence over time (Cooper et al., 2011; Blaschke et al., 2012; Demonceau et al., 2013). For example, a study analyzing medication adherence patterns of nearly 17,000 patients over a 1-year period revealed a gradual decrease in optimal implementation adherence by nearly 35% with approximately 40% of patients discontinuing their treatment (Blaschke et al., 2012). Another study analyzing dosing histories for hypertensive patients found that 50% of patients stopped the medications after 1 year and nearly 95% missed a dose in the year (Vrijens et al., 2008).

In terms of the effect of the pharmacist intervention, there was an increase in average PDC rates for all of the drugs after the intervention. These results align with findings from randomized controlled trials where medication adherence increases after a pharmacist intervention (Al-Jumah and Qureshi, 2012; Pousinho et al., 2016). A systematic review of interventions to improve medication adherence stated that counseling provided by health care professionals, such as pharmacists, could be not only effective but also cost-beneficial in improving medication adherence (Nieuwlaat et al., 2014). Also, face to face interventions, like the ones provided in community pharmacies, have a positive impact on enhancing medication non-adherence (Conn et al., 2016). Despite this amount of evidence, real-life effectiveness of these interventions once the evaluation phase is over remains unknown. Observational studies of implemented interventions, which usually rely on big data sources of patient registries and health records, are essential to determine whether patients in real-life practice are achieving the expected outcomes in a wider and more representative population. This implies they are crucial to assess the translatability of the results obtained in randomized controlled trials, providing key stakeholders like policy-makers evidence to support health care policies and funding allocation. Nevertheless, our study findings on real practice settings follow similar trends to those reported in randomized controlled trials.

The analysis of dispensing records after the pharmacist intervention showed an 8% decrease on average PDC 12 months after the intervention was delivered. Similar to our results, a recent meta-analysis found a 1.1% decrease in the effect of adherence interventions per month of follow-up, suggesting their impact tends to decline over time (Demonceau et al., 2013). Similarly, the number of adherent patients (PDC >= 80%) declined 1 year after the intervention. These results also align with previous evidence showing a diminution in the number of adherent patients to different chronic medications over time (Blaschke et al., 2012; Keyloun et al., 2017). This may suggest a need for continuous adherence interventions and sustained follow-up integrated into the patient’s treatment plan. This would allow not only the identification of barriers in non-adherent patients, but also the monitoring of current or new risk factors in patients showing optimal adherence and the development of tailored strategies to minimize their impact. Adherence interventions and more continuous follow-ups can be implemented in standard community pharmacy dispensing practice. Community pharmacy is an ideal place to continue to evaluate and discuss adherence with a patient over time due to patients returning, often monthly, for their repeat prescriptions. In fact, pharmacists have been found to have a positive impact on medication adherence in different clinical conditions (Taitel et al., 2012; Pousinho et al., 2016).

With the majority of patients not reaching the common threshold of a PDC of 80%, there remains opportunity for improvement. Often, single component interventions only affecting one aspect of non-adherence are minimally effective (Choudhry et al., 2009). Medication non-adherence is a complex and multifactorial problem influenced by multiple determinants across different domains (Vrijens et al., 2012; Kardas et al., 2013). This might be the reason why complex and multicomponent interventions are often seen as the most effective strategies for improving adherence. Potential approaches to improve the current MedScreen Compliance GuildCare adherence intervention might include the use of the perceptions and practicalities approach, distinguishing between unintentional and intentional non-adherence (Horne et al., 2005). This would allow a more tailored approach to the problem, increasing the likelihood of success. Intervention for patients presenting unintentional non-adherence may target practical barriers through more technical components (i.e., interventions providing any gadget, instrument, or system that facilitate the medication intake or increase convenience of the medication taking process). Some examples include helping patients to adopt routines of medication taking trough SMS reminders or alarms (Vervloet et al., 2012; Thakkar et al., 2016). In contrast, intentional non-adherence is related to perceptual factors like lack of motivation or beliefs toward the medication therapy (Horne et al., 2005). Interventions for patients with intentional non-adherence may consider targeting behavioral intention based on modifying patient’s attitudes and beliefs through the use of evidence-based frameworks such as the necessity and concerns framework (Clifford et al., 2008) or motivational interviewing (Levensky et al., 2007). A combination of both of the above mentioned scenarios might also be possible, requiring interventions with multiple components (Nieuwlaat et al., 2014). In a recent network meta-analysis, multicomponent interventions were found to have the most effective long-term improvement on adherence.

The conservative estimates of using PDC, averaging approximately 67%, produced well below considered “adherent” rates in patients, generally accepted at 80% or greater (Sodihardjo-Yuen et al., 2017). From previous research, PDC has been affirmed to be a more accurate and conservative representation of adherence compared to MPR (Martin et al., 2009). This allows the suggestion that while a MPR monitoring in real-time is helpful, a PDC calculation may be more valuable as the latter accounts for overlapping days and medication switch, two very likely conditions to happen in these community pharmacies. Therefore, measurement of medication adherence can be more consistent and accurate in this particular setting and a better intervention can be provided.

There were some limitations to this analysis. Dispense records were only associated to the pharmacy where patients were intervened. If the patient claimed a medication in a different pharmacy, this data was not recorded in this database. Because of this, it is not possible to know if patients actually discontinued their treatment. This is why only implementation adherence was reported, accounting from the first to the last available dispensing record. However a sensitivity analysis was performed on patients claiming all dispensing’s in the affiliated pharmacies. Additionally, while these results showed an improvement in adherence implementation shortly after the intervention was performed, we must also crucially consider the variability of the intervention between pharmacists and pharmacies. As this was retrospective data, no fidelity measures were able to be used to understand the full extent of the execution of these adherence interventions. Conversely, this could be found as a strength of the study as this was real-life practice with no trial variables impacting the results. At the very least, these interventions cause a pharmacist to alert a patient when they are seemingly non-adherent. Feedback interventions similar to this has shown success in other studies and meta-analyses, questioning if the feedback or the actual educational approach of the intervention is the most effective (Demonceau et al., 2013). To our knowledge, this is the first study utilizing big data analysis techniques to determine the effectiveness of a community pharmacy intervention in a real-life setting in Australia. Future research in this area could further explore on the determinants of PDC decreases over time.

## Conclusion

Integration of big database analysis techniques of dispensing records from community pharmacies across Australia provided results on implementation adherence before and after a pharmacist intervention within usual practice. Sub-optimal implementation adherence is a prevalent problem with the average PDC decreasing over time. An increase on average PDC was observed after the intervention, with a steady decline over time for each one of the drugs analyzed. Establishing follow-up mechanisms, enhancement of the intervention using an evidence based approach and incorporating a more accurate method for the real time analysis of dispensing data by using big data techniques would assist community pharmacists in improving medication adherence.

## Author Contributions

VG-C, SB, AT-R, EW, and RC contributed to the design of the study. BD organized the database and contributed to data analysis. AT-R performed the data analysis. AT-R, EW, and VG-C wrote the first draft of this manuscript. VG-C, SB, AT-R, EW, RC, BD, and FF-L contributed to manuscript revision, read and approved the submitted version.

## Conflict of Interest Statement

The authors declare that the research was conducted in the absence of any commercial or financial relationships that could be construed as a potential conflict of interest.
